# Revalidation and rationale for high pKa values of unconjugated bilirubin

**DOI:** 10.1186/1471-2091-8-7

**Published:** 2007-05-02

**Authors:** J Donald Ostrow, Pasupati Mukerjee

**Affiliations:** 1 Gastroenterology Section, Department of Medicine, Northwestern University Medical School, 310 E. Superior Street, Chicago, IL 60611, USA; 2 Research Service, D.V.A. Lakeside Medical Center, 333 E. Huron St., Chicago IL, 60611, USA; 3 Current address : GI/Hepatology Division, Box 356424, Univ. Washington Medical Center, 1959 NE Pacific Street, Seattle, WA 98195-6424, USA; 4 School of Pharmacy, University of Wisconsin, 777 Highland Ave., Madison, WI, 53705-2222, USA

## Abstract

**Background:**

Our prior solvent partition analysis, published in 1992, yielded pKa values for unconjugated bilirubin of about 8.1 and 8.4, but these results have been challenged and studies by other methods have suggested pKa values below 5.0.

**Methods:**

We repeated our published solvent partition studies, using ^14^C-unconjugated bilirubin highly purified by extraction of residual labeled impurities from CHCl_3 _into an aqueous buffer, pH 7.0. Partition ratios at six pH values from 5.0 to 9.0 were determined by radioassay and compared with our prior values obtained by diazo assay.

**Results:**

At pH values ranging from 4.8 to 9.2, stable aqueous/chloroform ^14^C-partition ratios did not differ significantly from our published partition ratios based on diazo assay.

**Conclusion:**

These results support the high pKa values of unconjugated bilirubin, above 8.0, derived from our earlier solvent partition study. In both studies, our measurements were based on the rapid analysis of clearly under-saturated solutions of highly-purified bilirubin over a wide pH range, using properly purified and preserved solvents. No previous direct estimate of the aqueous pKa values of unconjugated bilirubin meets all these preconditions. Three theoretical factors acting in combination, each related to the unique, extensive internal H-bonding of the -COOH groups, are proposed to support high pKa values of unconjugated bilirubin in water: a) donation of an H-bond from the -OH moiety of the -COOH group, which is broken on ionization; b) hindered solvation of the -COO^- ^group after ionization; and c) restricted rotation of the -COO^- ^and -COOH groups. Our findings and rationale rebut methodological and theoretical criticisms leveled against our prior work. High pKa values for unconjugated bilirubin dictate that: a) bilirubin diacid, which readily diffuses across membranes and can cause neurotoxicity, is the dominant unbound bilirubin species of unconjugated bilirubin in plasma at physiological pH; b) at the near-neutral pH range of gallbladder bile, the monoanion is the major unconjugated bilirubin anion present, concordant with the finding that the calcium bilirubinate precipitated in gallstones is the monoanion salt. Our conclusions are thus relevant to understanding bilirubin-induced neurological disease in severely jaundiced neonates and the precipitation of calcium bilirubinate salts in gallstones.

## Background

In solution, unconjugated bilirubin (UCB) exists as three species in equilibrium, the fully protonated diacid, the monoanion, and the dianion [1]. These three UCB species have quite different properties and functions [1]. The true pKa values of UCB are of great physiological and well as basic relevance, because they affect UCB species distributions [1] and estimates of the aqueous solubility of UCB diacid [2]. Unfortunately, there are tremendous variations among the reported pKa values for bilirubin in aqueous solutions, as determined by a wide variety of methods. Most studies in the literature suggested pKa values below 7.0 and even below 5.0, whereas our solvent partition studies [3] indicated that the two pKa values were higher, 8.12 and 8.44. If, in contrast, the assumed pKa values of 4.4 and 5.0 [4] are used to represent low pKa's, the ratio of diacid/dianion at pH 7.4 would change from 0.58 (high pKa's) to only 4 × 10^-6 ^(low pKa's), and the solubility of UCB diacid would change from the experimental value of 5 × 10^-8 ^M [3] to less than 10^-14 ^M [2]. Such differences are clearly of great significance in understanding the interactions of UCB and its pathophysiological effects, most notably: a) bilirubin-induced neurological disease in severely jaundiced neonates [5,6]; and b) the precipitation of calcium bilirubinate salts in gallstones [7].

The reported variations in pKa estimates are due in large part to the methodological difficulties of studying bilirubin at concentrations below its low aqueous solubility limit (< 0.1 *μ*M at pH ≤ 7.8 [2]) and the ready degradation of the pigment to more polar derivatives with much higher solubility and different ionization properties [8-10]. The tendencies of UCB to deteriorate require that the pigment be purified just before experimental use, and that the measurements be made over a brief time span. Recently, we have shown that the customary purification method [11], leaves a small but significant proportion of polar impurities, most of which can be removed by serial extraction into aqueous buffer at pH 7.0 [10]. The present work, utilizing ^14^C-UCB highly purified by this new approach, permitted us to determine, by direct radioassay of the two phases, the ^14^C-partition ratio at very low aqueous concentrations of UCB that were uniformly below saturation. The results confirmed our findings based on solvent partition of unlabeled UCB with diazo-assay of the two phases [3], further supporting our conclusion that the pKa values of UCB are both above 8.0.

## Methods

### Labeled UCB

^14^C-UCB (spec. act. 8466 dpm/*μ*g) was prepared by biosynthetic labelling from 4- ^14^C-δ-aminolevulinic acid in bile-fistula rats, with isolation of ^14^C-UCB from the bile as described elsewhere [12]. After alkaline extraction of some impurities from UCB in chloroform, followed by recrystallization [11], the further impurities were removed by serial extraction from chloroform with 0.1 M phosphate buffer, pH 7.0 [10]. These purified chloroform solutions of ^14^C-UCB had extinction coefficients of 61.2 mM^-1 ^cm^-1^ at 453 nm and, by thin-layer chromatography [11], contained over 94% of the IXα-isomer and only 0.03% of ^14^C- in non-bilirubin compounds.

### Solvents and buffers

Distilled water and argon (Amerigas, Valley Forge, PA) were purified as described previously [10]; the argon was then presaturated with purified chloroform and water. Reagent-grade chloroform (J.T. Baker, Phillipsburg. NJ) was purified further as described [3,10,13] and stabilized by storage under purified water and argon in brown glass bottles in the dark for less than 72 hr before use [3,10]. All other compounds were reagent grade, obtained from Baker or Sigma. Buffers used (0.1 M sodium salt) were: citrate at pH 5.0 and 6.0, Pipes or phosphate at pH 7.0, Hepes or phosphate at pH 7.4, Hepps or phosphate at pH 8.0, and borate at pH 9.0; the final ionic strength was adjusted to 0.15 with addition of NaCl.

### Solvent partitions

These were performed in dim light under an argon atmosphere, as we described previously [3,10]. Purified ^14^C-UCB, dissolved in 2.0 mL purified chloroform at concentrations of 0.6 to 0.8 mM, was serially partitioned at 25 ± 1°C, with 40 mL of buffer that had been pre-equilibrated with purified chloroform. After centrifugation for 2 min. at 1,350 × *g*, the pH of the upper phase was measured with an Orion Research model 901 digital pH-meter and glass electrode, and measured aliquots of chloroform and aqueous phases sampled. All remaining upper phase and any interphase were then removed, an identical volume of aqueous buffer layered on the residual lower phase, the tube again deoxygenated with argon, and the partition repeated serially for another 4 cycles, using fresh aqueous buffer for each cycle.

### Assays

Radioactivity in 1.0 to 2.0 mL upper (Bw) and 10 to 25 *μ*L lower (Bc) phases, added to 10 mL of scintillation cocktail (Ecolite™(+), ICN Biomedicals, Irvine, CA), was measured in a Beckmann LS8000 liquid scintillation spectrometer, with correction for quenching using external standards (^14^C Quenched LSC Standards, DuPont-New England Nuclear). Specific activities of the ^14^C-UCB were measured at the start and conclusion of each set of partitions by concomitant radio- and diazo-assay [14].

### Statistical analyses [15]

It was determined if the individual ^14^C-PR values from pH 4.8 to 9.2 fell within the 95% confidence limits of the PR_*diazo *_values, delimited by the log PR_*diazo *_values ± 0.382 (± 2 × the [root mean sq. deviation]^0.5 ^of the log PR_*diazo *_values [3]).

## Results

### Changes in ^14^C-PR over serial partition cycles

As described previously [10], similar results were obtained at pH 6.0, 7.0 and 7.4. The ^14^C-PR declined steeply between the first and second cycles, to 40–67% of the initial ^14^C-PR, and essentially stable, lower ^14^C-PR were attained from the second cycle on. By contrast, at pH 8.0 and 9.0, the decline between cycles was shallow and not significant. There were no significant differences in ^14^-PR between phosphate and zwitterion buffers at pH 7.0, 7.4 or 8.0.

### Comparison of ^14^C-PR with PRdiazo

**Figure 1:**
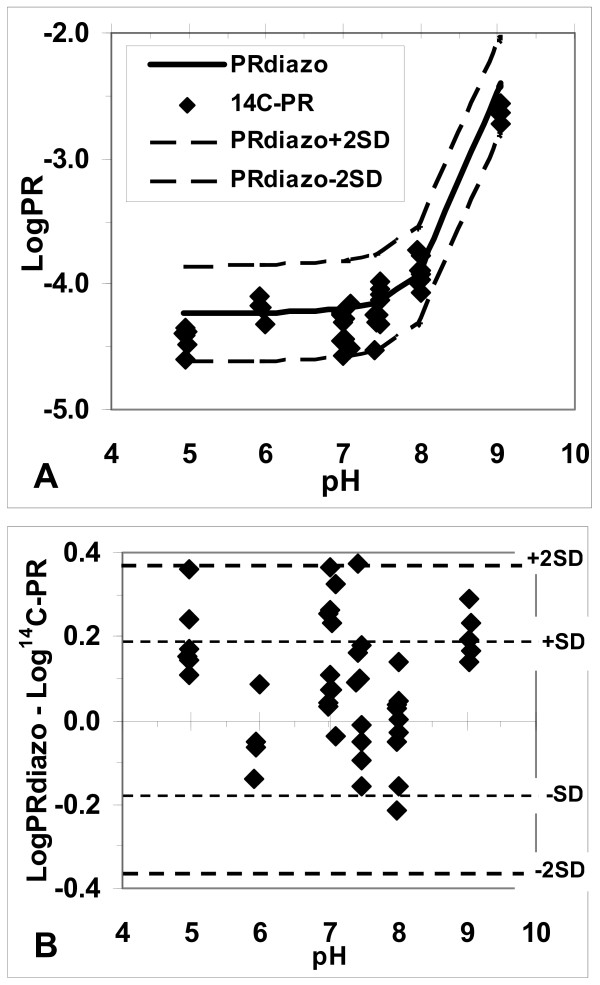
**Comparison of aqueous:chloroform partition ratios of radioactivity (^14^C-PR, ◆), using ^14^C-UCB, with partition ratios of unlabeled UCB (PR_diazo_)**. PR_diazo _was calculated from the equation derived from diazo-assay of the two phases [3], plotted against pH of the aqueous buffer. Panel A – Log PR vs. pH. Heavy solid line shows the PR_diazo _derived from computer modeling the diazo-based data [3]. Panel B – The same data, plotted as the difference, log PR_diazo _– log ^14^C-PR. In each panel, the heavy dashed lines represent the 95% confidence limits of the log PR_diazo _data, equal to 2 × 0.191, the [root mean sq. deviation]^0.5 ^of the log PR_diazo _data [3]. Only one of the ^14^C-PR data points (at pH 7.4) falls barely outside the 95% confidence limits for the PR_diazo _data.

Partitions performed with an upper to lower phase volume ratio of 20:1 yielded reproducible low ^14^C-PR from the 2^nd ^through 5^th ^cycle at all pH values studied, as described previously [10]. In Fig. 1A, the logarithms of these stable ^14^C-PR values are plotted against the measured aqueous phase pH values and compared with the computer-derived curve of log PR vs. pH, obtained by diazo assay of phases from partition of unlabeled UCB [3]. All the log ^14^C-PR values but one fall within the mean ± 2 SD (± 2 × 0.382) of the log PR_diazo_. Fig. 1B plots the difference between the log PR_diazo _and log ^14^C-PR against pH. The mean difference of 0.094 ± 0.146 was not significantly different from zero. Again, only one point barely falls outside the 95% confidence limits.

## Discussion

### Purity and stability of ^14^C-UCB

As described previously [10], after the first partition cycle, less than 0.03% of labeled contaminants partitioned from the highly purified ^14^C-UCB used in these studies. Degradation of the UCB was minimized by the rapidity of the partitions and exclusion of light and oxygen, which are promoters of bilirubin oxidation [8].

### Role of chloroform purification method and possible lipid contaminants

Boiadjiev, *et al*. [16] had reported the formation of UCB aggregates in some CHCl_3 _solutions when pure distilled CHCl_3_, exposed to Parafilm^® ^or plastic caps, was used as solvent. They suggested that "adventitious lipid contaminants can cause aggregation in CHCl_3_" and that this may be relevant to our CHCl_3_-water partition studies [3].

When CHCl_3 _is distilled after some careful washing and drying, removal of the ethanol stabilizer results in oxidative degradation of CHCl_3_after minimal exposure to light and air [17]; the resultant phosgene (carbonyl chloride), chlorine, and HCl cause rapid degradation of UCB [18]. In our procedures [3,10], CHCl_3_, after vacuum distillation, was extracted with 0.1 M H_2_SO_4_, 0.1 M NaOH, and then five times with purified water. Our chloroform was not only thus purified, but was also stored for less than 72 hr in the dark, in an argon atmosphere, under a layer of purified water, which stabilizes the CHCl_3 _against regeneration of the oxidative products [17]. Water-stabilized CHCl_3 _has been used successfully for quantitative analysis of anionic and cationic lipids in water at concentrations as low as 10^-6 ^M, by their nearly complete extraction as ion pairs with cationic and anionic dyes [13,19,20]. In those studies, no serious impurities were noted in the blank values, rendering it unlikely that significant lipid impurities were present in the similarly-prepared CHCl_3 _that was utilized in our present and prior [3] partition studies with UCB. As noted previously [3], the lack of significant change in PR over a range of Bc from 0.2 to 1.0 mM mitigated against the possibility of significant self-aggregation of UCB in the CHCl_3 _phase, contradicting suggestions [16] that this could have occurred in our studies.

Partition methods, using water-stabilized CHCl_3_, have been used to study successfully the self-association of methylene blue in water [21] and to determine, with high precision and accuracy, the pKa values of two organic acids in water [22]. These studies with other compounds validate use of this procedure to evaluate these properties of UCB, as long as degradation of the pigment is minimized. Our use of dim light and rapid extraction procedures, in acid-washed pyrex glass partition tubes sealed under argon with tight-fitting, inert Teflon-lined screw caps, further minimized chemical alterations in the CHCl_3 _as well as the UCB.

### Comparison of partition ratios obtained by ^14^C- vs. diazo-assay

At pH values from 4.8 to 9.0, the reproducible, minimum ^14^C-PR of radiolabeled UCB, obtained after multiple partition cycles, fell within the 95% confidence limits of the PR_diazo _values we reported previously [3], using diazo-assay of the two phases after only two partitions. In that prior study [3], the aqueous phase concentration of UCB was determined by back-extraction into a small volume of chloroform, permitting diazo-assay of the UCB thus concentrated; no significant differences were detected between PR_diazo _obtained on the first and second cycles. By contrast, in the present work, ^14^C-PR was uniformly higher in the first than in the second cycle, due to partition of labeled polar impurities into the aqueous phase. Direct analysis of the polar HPLC peaks had demonstrated that these polar impurities were not diazo-reactive [10]. In addition, due to their high PR, these impurities would be poorly back-extracted into chloroform and thus little detected, even if they were diazo-positive. These factors account for the previously-reported correspondences between PR_diazo_ for the first two cycles [3].

The HPLC analyses demonstrated that the only diazo-reactive product in the aqueous phase was UCB [10], accounting for the agreement of the PRdiazo with the ^14^C-PR. The mean difference between PR_diazo_ and ^14^C-PR was slightly positive and not significantly different from 0 (Fig. 1B); had there been a significant contribution of labeled derivatives of UCB in the upper phase, the ^14^C-PR should have significantly exceeded the PR_diazo_. The concordance of the present ^14^C-PR with our previously-reported PR_diazo _values for UCB [3] validates our prior data and the derived pKa values of 8.12 and 8.44.

### Comparative evaluation of experimental pKa values for bilirubin

A wide variety of mean pKa values for UCB have been reported, ranging from 4.4 to 8.3 [23,24]. We believe that estimates of the aqueous pKa values of UCB in a mono-molecular state are meaningful only if two thermodynamic conditions are satisfied: (1) Solutions used must avoid serious supersaturation to prevent irreversible aggregation of UCB; and (2) account must be taken of any reversible self-association, particularly those of UCB dianions [3,25]. In addition, the facile degradation of UCB calls for experiments done over a very short time span, under anaerobic conditions, with minimal exposure to light, using highly-purified UCB [8,10].

In our partition experiments, reported previously [3] and confirmed here, true solutions in CHCl_3 _were used to determine water/CHCl_3 _PR values, thus avoiding supersaturation effects. All PR determinations were conducted in less than 40 minutes, thus minimizing UCB degradation. Along with the pKa values of 8.12 and 8.44, a dimerization constant for the dianion, 0.26 *μ*M^-1^, was determined to account for its reversible aggregation. Our log PR increased by only about 0.3 as the pH changed from 5.0 to 8.0, as expected from these high pKa values. Low pKa values, e.g. 4.4 and 5.0 assumed by Overbeek, *et al*. [4], would necessitate that log PR increases by more than 5.65 units from pH 5 to 8, requiring deviations from our data by up to 30 times their S.D. values. Our partition data [3], confirmed here, are, therefore, seriously inconsistent with such low, assumed, pKa values.

As regards experimental pKa values in the literature, we are not aware of any other study in water in which systematic equilibrium measurements were carried out below a total aqueous UCB concentration of 0.1 *μ*M over the pH range of 5.0 to 7.8. Such low concentrations pose serious experimental difficulties and analytical challenges, but must not be exceeded by much if supersaturation is to be avoided [2]. This pH range provides a base line for our high pKa values, and is clearly of crucial importance if pKa values are about 5.0 or lower. Studies conducted only at high pH values and/or with highly supersaturated concentrations of UCB are not interpretable unless any reversible or irreversible self-aggregation is fully accounted for. Such studies will not be discussed here.

Based on extrapolation to 0% DMSO from ^13^C-NMR measurements in mixtures of DMSO and water, the two pKa values of mesobilirubin in water have been reported to be 4.2 and 4.9 [24]. Due to the demonstrated problems of insolubility [2], large errors in pH measurements in the mixed solvents [26,27], and the long, even overnight duration of the ^13^C-NMR analyses [23], these studies are also not interpretable. We conclude that no reliable pKa value of UCB, in an unaggregated, molecular state, has been directly determined in water for comparison with our partition-derived pKa values of 8.12 and 8.44 [3], confirmed here.

### Rationale for high pKa values for UCB

**Figure 2:**
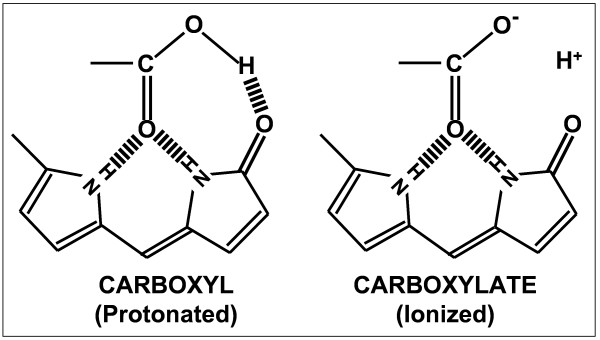
**Two-dimensional representation of the structural environment of the carboxyl groups of unconjugated bilirubin**. Left panel shows the three internal hydrogen bonds (||||||) that involve each -COOH group of unionized UCB diacid. All the donors and acceptors of the hydrogen bonds belong to the UCB molecule. With ionization of the -CO-**OH **group (right panel), the H-bond involving the -OH group is lost, but the two H-bonds remain that involve the carbonyl group of the carboxylate anion (-**CO**-O^-^).

If there were no intramolecular hydrogen-bonds in UCB, the pKa values would be expected to be similar to the values of 4.4 and 5.0 assumed by Overbeek *et al*. [4]. In UCB, however, each -COOH group is involved independently in three internal hydrogen bonds [28], one of which is broken with ionization [1] (Fig. 2). This unique pattern of three H-bonds to each -COOH group and two remaining H-bonds to each ionized -COO^- ^group in UCB produces a crowded and constrained microenvironment for both groups and restricts their mobility, as is made clear by a space-filling molecular model of UCB dianion [29]. We ascribe the high pKa values of UCB to at least three effects arising from these H-bonds: a) donation of an H-bond from the -OH moiety of the -COOH group; b) hindered solvation of the -COO^- ^group; and c) restricted rotation of the -COOH and -COO^- ^groups, which also contributes to suboptimal solvation of these groups. Since we have found no model system that replicates, even approximately, the complex H-bonding of UCB, our discussion is necessarily based on various pKa values from the literature, for which these three factors have been invoked individually and found necessary for interpretation.

#### a) The effect of breaking the -O-H **||||||** O=C< bond on ionization

When a -COOH group acts as a donor of an H-bond, which is cleaved upon dissociation of the proton, the pKa is raised, because the H-bond stabilizes the -COOH group with respect to the -COO^- ^anion. For example, in neutral molecules, such as macrocyclic polyethers [30,31], pKa values of the -COOH groups can be elevated by as much as 1.5 units [30]. Similarly, in many dicarboxylic acids, the observed high pKa_2 _values have been ascribed, in part, to breaking, upon dissociation, of the H-bond donated by the -COOH group in the monoanion [32-34]. McCoy [33] applied the method suggested by Westheimer and Benfey [35] to estimate intramolecular H-bonding effects on the elevation of pKa_2 _values in water of maleic acid and several other 1,2 dicarboxylic acids with fairly rigid skeletons. The results varied over a wide range, from about zero for phthalic acid to about 2 for furan-3,4-dicarboxylic acid and cyclobutane-1,2-dicarboxylic acid. Such differences were attributed to subtle geometrical factors involving the spacing of the carboxyl groups and the rigidity of the molecular skeleton to which the -COOH groups are attached [33]. These remarkable differences between molecules of similar structure underscore the hazards of applying molecular model systems to estimate effects of H-bonding on pKa values.

Such considerations may be important for UCB. Among other factors, the presence of two H-bonds, anchoring the >C=O group of each dissociating -COOH group in UCB (Fig. 2), is likely to more optimally position the same -COOH group to donate an H-bond to the pyrrolic -NH-CO- group in the opposite dipyrrole half of the molecule (Fig. 2). In addition, the formation of the three H-bonds with each -COOH group is likely to involve a significant co-operativity and a lower loss in entropy when compared to the H-bonding of a freely-rotating -COOH group. Both pKa values of UCB are, thus, expected to be raised because of the H-bond donated by each of the two -COOH groups. Magnitudes are difficult to predict but an elevation of 2 or more units cannot be ruled out on *a priori *grounds.

Others have expressed a contrasting view that H-bonding can *decrease *pKa values in UCB [16,23,24,36]. Two types of model systems have been used for these arguments; we believe they are inappropriate. The first is the reduction in the pKa_1 _of dicarboxylic acids, such as maleic acid [34,35], which has been ascribed in part to the stabilization of the monoanion by the formation of an H-bond (-O-H |||||| O^-^) between the -COOH and -COO^- ^groups after the first ionization [33,35]. In sharp contrast, in UCB, an H-bond is cleaved at each ionization step and *no new H-bond is formed*, rendering the comparison invalid. An example of the second type of model system used is methyl phthalate. That its pKa (3.25) is lower than that of benzoic acid (4.20) has been ascribed entirely to intramolecular H-bonding in methyl phthalate [24]. We note that a much larger decrease in pKa can be seen between acetic acid (4.8) and a monoester of oxalic acid (1.5) [37]. The argument is without merit, however, since it has been shown clearly that there is no intramolecular H-bonding in the monoanions of either phthalic acid or oxalic acid [35], rendering it extremely unlikely that there is any such H-bonding in their monoesters. Moreover, it has been shown clearly [35] that any internal H-bonding in a monoester (such as methyl phthalate) must *increase *its pKa from the value in the absence of H-bonding. Other factors, particularly the well-known electrostatic effects of dipoles, such as those of carbalkoxyl groups [38], provide better explanations. We have found no system for which, upon its dissociation, the breaking of an H-bond donated by a -COOH group causes a decrease in pKa. It is certainly hard to justify, on theoretical grounds, that an H-bond enhances the dissociation of a -COOH group.

#### b) Steric hindrance to solvation

The extensive H-bonding involving both the -COOH and -COO^- ^groups of UCB places them in an unusually crowded environment, where solvation by water must be inhibited because of restricted space and poor accessibility. Steric inhibition of solvation is expected to increase the energy of a charged species much more than an uncharged one, so that sterically-hindered carboxyl groups are expected to have higher pKa values [39]. For example, the pKa of acetic acid can be raised by 1.4 units or more by attachment of multiple bulky, uncharged alkyl groups, as in methyl-t-butyl-neopentyl acetic acid [40,41]. These increases in pKa values have been ascribed to hindered solvation, particularly of the -COO^- ^group [39-42]. Steric hindrance to solvation has also been suggested to account for 2.2 to 6.4 unit increases in pKa values of pyridine and aniline highly substituted with t-butyl groups [43].

The importance of the interactions of the -COO^- ^ion arises from the high values of its solvation energy. In units of kcal/mol, the lattice (sublimation) energies of NaCl and CH_3_COONa are 189 and 193 respectively, the corresponding enthalpies of solution in water being 0.9 and -4.1 [44]. The calculated solvation energies are -188 kcal/mol for NaCl and -197 kcal/mol for CH_3_COONa. Using the estimate of -106 kcal/mol for Na^+ ^[42], the estimated values of -82 and -91 kcal/mol for Cl^- ^and CH_3_COO^- ^indicate the great strength of ion-solvent interactions. Compared to these, fairly strong H-bonds involve about 5 kcal/mol [34]. The free energy change for UCB associated with a rise in pKa_1 _from 4.4 [4] to 8.1 [3] can be estimated from the change in -RTlnKa_1_, where R = 1.987 cal.deg^-1^mol^-1^, and T is 298 K; it is given by -2.303 RT(8.1–4.4) = ~5 kcal/mol. Thus, even a small, fractional change in the high solvation energy of a -COO^- ^group, arising from hindered solvation, can produce large effects on pKa values. It is not known whether the hindered solvation in the constricted, crowded microenvironment of the -COO^- ^groups in UCB can produce larger effects than alkyl substitutions in acetic acid, but this cannot be ruled out.

#### c) Restricted rotation of the -COOH and -COO^- ^groups

Free rotation around the C-COOH or C-COO^- ^bond allows these groups, particularly the -COO^- ^group, to adapt to optimal solvation shells containing many water molecules. In UCB, the two H-bonds to the >C=O group must restrict the free rotation of the -COOH group, and, more importantly, of the -COO^- ^group as well. Constrained rotation of the -COO^- ^ion is shown, for example, by molecular models of t-butyl malonic acid [45]. Such restricted rotation makes it difficult for the solvation shell, which usually includes several solvent molecules, to achieve the optimum geometry as compared, for example, to the freely-rotating -COO^- ^group of propionic acid.

In a useful summary, McCoy notes that crowded microenvironments, leading to restricted rotation effects, sometimes combined with hindered solvation, can increase by several units the pKa_2 _values of many substituted dicarboxylic acids [33]. The systems include: t-butyl malonic acid (mentioned above) [45], racemic di-t-butyl succinic acid [46], some disubstituted malonic acids [47], and some substituted cyclopropane-1,2-dicarboxylic acids [33,48,49]. The intramolecular hydrogen bonding of the -COOH and -COO^- ^groups of UCB (Fig. 2) is likely to be very effective in restricting their rotation. The consequent poor solvation can destabilize the -COO^- ^groups in UCB, causing a significant increase in pKa values.

## Conclusion

The present study with ^14^C-UCB has revalidated our prior solvent partition study, based on diazo assay of unlabeled UCB [3], and rebutted the methodological criticisms leveled at that study. In addition, we have proposed three factors, related to the internal hydrogen bonding of UCB, that, acting in concert, could reasonably be expected to increase the pKa values of the -COOH groups of UCB by about 3.5 units above the values expected for propionic acid, for which none of the three factors is operative. These rationales respond to the repeated assertion by others that there is "no precedent for such a large increase in the pKa of carboxyl groups due to internal hydrogen bonding". The complexity of the structure of UCB (Fig. 2) and the interactions discussed above, however, preclude a simple, quantitative prediction of the pKa values of UCB, or the relative importance of the three factors in affecting its ionization. Only the H-bond between the CO-OH and pyrrolic O=C-NH, groups (Fig. 2) has been discussed before in any detail for interpreting pKa values [3,16], but we do not believe that the other two factors can be ignored. We also note the possible importance of cooperativity involved in the formation of the trio of H-bonds to the -COOH group and the possible role of the H-bonds donated to the carboxylic >C=O groups in affecting the strength of the -CO-OH**||||||**O=C-NH bonds through improved geometrical fitting. Additionally, effects of the hindered solvation and restricted rotation on -COO^- ^groups of UCB might be mitigated by the weakening or even breaking of the two -N-H**|||||**O=C< bonds attached to each carboxylate group (Fig. 2). These effects are likely to counter some expected strengthening of the H-bonds to the >C=O moiety of a -COO^- ^group as compared to that of a -COOH group [28,50], possibly leading to conformational changes in the UCB molecule on ionization, as suggested previously by Carey and Spivak [29]. A significant fraction of the dianions might even be present in a non-H-bonded form, a representation sometimes used for the dianion [51,52]. Such changes may have important effects on the binding and self-association properties of the dianions [1].

Since the literature contains many reports, utilizing a wide variety of methods, which favor pKa values for UCB below 7.0 and even below 5.0, we are preparing an extensive review concerning the validity of the methods used in those experiments. The critique will focus especially on the purity of the UCB used, the presence of supersaturation with UCB in the systems studied, and extrapolation from results in non-aqueous solvents [53,54]. The review will also include a critical discussion of the extremely important role of pKa values of UCB in determining its pH-dependent interactions with phospholipid vesicles [1,55], bile salts [56,57] and cyclodextrins[58].

## List of Abbreviations

DMSO, dimethylsulfoxide; H-bonding, hydrogen bonding; PR, aqueous/chloroform partition ratio; ^14^C-PR, PR of radioactivity; PR_diazo_, PR of diazo-reactivity; UCB, unconjugated bilirubin.

## Authors' contributions

JDO performed the partition studies. PM conceived of the study and performed the mathematical modeling and statistical analysis of the data. Both authors participated in the design of the study, drafted the manuscript, and read and approved the final manuscript.
